# ASO Author Reflections: Clinical Outcome After Upfront Curative-Intent Local Treatment of Metastases in Patient with Deficient Mismatch Repair Versus Proficient Mismatch Repair Metastatic Colorectal Cancer

**DOI:** 10.1245/s10434-023-14069-z

**Published:** 2023-08-10

**Authors:** Koen Zwart, Jeanine Roodhart

**Affiliations:** Department of Medical Oncology, University Medical Center Utrecht, Utrecht University, Utrecht, The Netherlands

## Past

Local therapy of metastases prolongs overall survival and is recommended for all patients with resectable metastatic colorectal cancer (mCRC).^1^ However, mCRC is a heterogeneous disease and the subgroup of deficient mismatch repair (dMMR) tumors is biologically different compared with proficient mismatch repair (pMMR).^2^ It is unclear whether curative-intent local therapy of metastases is of similar benefit for patients with dMMR mCRC compared to pMMR patients. Knowledge of clinical outcomes after local treatment for dMMR mCRC patients is of particular importance to guide shared decision-making given the new treatment option of immunotherapy for dMMR mCRC with durable responses and good overall survival.^3^

## Present

This Dutch, observational, nationwide study compared 84 patients with dMMR mCRC who received curative-intent local therapy of metastases with 1,099 patients with pMMR mCRC in the pre-immunotherapy era.^4^ Median recurrence-free survival (RFS) was longer in the dMMR mCRC group compared with the pMMR mCRC group (median 11.1 vs. 8.9 months) as well as 2-year RFS (43% vs. 21%). Baseline characteristics and type of local therapy differed between the groups. However, when adjusted for these variables in a multivariable analysis, dMMR status still demonstrated to be an independent predictor for improved RFS. These results align with three other exclusive cytoreductive surgery ± hyperthermic intraperitoneal chemotherapy studies, including 15 to 44 dMMR mCRC patients, also demonstrating superior RFS compared to pMMR.^5–7^

## Future

Based on the current evidence, local therapy with curative intent for patients with dMMR mCRC remains a valuable treatment option with a relative high proportion of patients who remain recurrence-free after 2 years. However, none of the present studies directly compare upfront immunotherapy versus upfront local therapy in patients with dMMR mCRC and resectable metastases. The choice between upfront local treatment versus upfront immunotherapy should be made by shared decision-making by weighing the chances of morbidity from local treatment versus the toxicity and adverse events of immunotherapy and the expected benefit for every individual patient. To better guide clinical decision-making, prediction models are needed to support which patients can be cured by local treatment alone and which patients will benefit most from immunotherapy. These data can be obtained from a combination of clinical trials and high-quality, real-world data and will improve personalization of treatment.
